# Nanosecond infrared laser (NIRL) for cutting roots of human teeth: thermal effects and quality of cutting edges

**DOI:** 10.1007/s10103-024-04173-1

**Published:** 2024-08-29

**Authors:** Reinhard E. Friedrich, Felix K. Kohlrusch, Thomas Ricken, Julian Grimm, Martin Gosau, Michael Hahn, Simon von Kroge, Jan Hahn

**Affiliations:** 1 Oral and Craniomaxillofacial Surgery, Eppendorf University Hospital, University of Hamburg, Martinistr. 52, 20246 Hamburg, Germany; 2 Section Mass Spectrometry and Proteomics, Center for Diagnostics, University Medical Center Hamburg-Eppendorf (UKE), Martinistr. 52, 20246 Hamburg, Germany; 3 Institute of Osteology and Biomechanics, Eppendorf University Hospital, University of Hamburg, Martinistr. 52, 20246 Hamburg, Germany

**Keywords:** Apicoectomy, Laser ablation, Micro-CT, Nanosecond infrared laser, Optical coherence tomography, Oral surgery, Thermal effects

## Abstract

A nanosecond infrared laser (NIRL) was investigated in cutting dental roots. The focus of the investigation was defining the preparation accuracy and registration of thermal effects during laser application. Ten teeth were processed in the root area using a NIRL in several horizontal, parallel incisions to achieve tooth root ablation as in an apicoectomy. Temperature change was monitored during ablation and the quality of the cutting edges in the roots were studied by means of micro-CT, optical coherence tomography, and histology of decalcified and undecalcified specimens. NIRL produced clearly defined cut surfaces in dental hard tissues. The automated guidance of the laser beam created regular, narrow dentin defects that tapered in a V-shape towards the ablation plane. A biologically significant increase in the temperature of the object and its surroundings did not occur during the laser application. Thermal dentin damage was not detected in histological preparations of treated teeth. Defined areas of the tooth root may be ablated using a NIRL. For clinical translation of NIRL in apicoectomy, it would be necessary to increase energy delivered to hard tissue and develop beam application facilitating beam steering for oral treatment.

## Introduction

Lasers are widely used in clinical and experimental settings for the treatment of oral lesions due to their precise tissue ablation capabilities [1]. The available tools for removing the root tip of diseased teeth with periradicular inflammation are limited to mechanical abrasion (drills) or shock wave transmission (piezo surgery) [2]. Both techniques ensure the targeted removal of the diseased tissue. However, when using these conventional tools the volume removed often exceeds the affected tooth root and bone. In contrast, laser application has the advantage of considerably more focused, refined tissue ablation. This can result in a smaller cavity that needs to heal and better stability of the preserved tooth in the socket. Although the use of lasers in dentistry and oral surgery is technically demanding and expensive, it offers the potential for preserving a larger portion of tooth root reducing the volume of root ablation. Moreover, the focused energy can easily lead to temperature increases, which can cause tissue damage [3–6]. Technical developments using highly energetic lasers with very short exposure times intend to an athermal, subcellular, explosive decomposition. This, based on the summation of the effects, allows for precise tissue removal [7–9]. The ablation principle is based on a novel approach to tissue processing by IR emitting light at a wavelength of 2940 nm, as described in [10]. With the referred conditions, the energy of the IR laser is effectively absorbed by water molecules and immediately converted into translational energy. The effect is caused by the vibrational motion of the hydroxy (OH) stretch band, which leads to a explosion of the water molecules. The ultrafast, subcellular energy transfer causes the decomposition of tissue and thus its transition into the gas phase [7], without heat transfer into the tissue. Conventional lasers, e.g. CO_2_, Er:YAG or Nd:YAG or lasers, rely other mechanisms or operate with much higher pulse energies, which leads to melting, burning or rough cutting edges [11–13]. However, an infrared (IR) picosecond laser also caused higher temperatures during experimental apicoectomy of teeth in a recent study, due to the hardness of tooth and a reduced water content compared to soft tissue [14].

In this study weinvestigated the potential of a nanosecond IR laser [10], tuned to the wavelength of 2940 nm, for precise ablation of the root of teeth. We focused on monitoring the surrounding tissue temperature to ensure that it remains at a biologically tolerable value during the procedure. Furthermore, we investigated the quality of the cutting edges and examined whether the volume of tissue removed is proportional to the duration of laser application.

## Materials and methods

### Nanosecond infrared laser (NIRL)

**Fig. 1:**
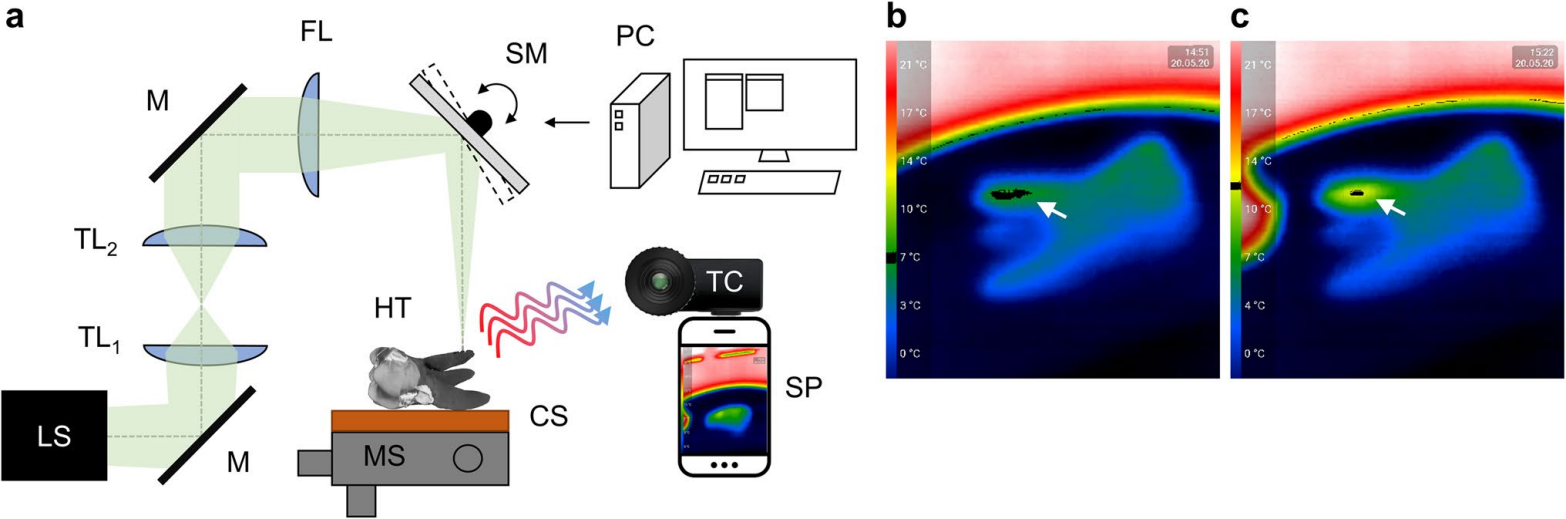
Experimental laser ablation setup (**a**). LS: nanosecond infrared laser system; TL_1/2_: telescopic lenses 1/2; M: silver mirror; FL: focusing lens (150 mm); SM: 2-axis scanning mirror; PC: controlling computer; HT: human tooth; MS: manual translation stage; CS: controlled cooling stage (-10°C); TC: temperature camera; SP: smartphone as readout device for the temperature camera. Images of the temperature camera before (**a**) and after (**b**) the NIRL ablation at the tip of the upper root (arrows). Temperature determination was achieved by adjusting a color threshold in the software Fiji [15] to the color-code of the ablation area

The experiments were carried out using a NIRL (Opolette SE 2731, Opotek, LLC, Carlsbad, CA, USA). The NIRL was used for serial incision of dental roots with a repetition rate of 20 Hz and 650 μJ at the focal point, measuring about 150 μm in diameter (FWHM). The standardized examination conditions for the ablation of hard dental tissue by infrared (IR) laser are described in detail elsewhere [14]. The basic laser ablation setup utilizing a NIRL is described in [10]. The experimental setup of this study is shown in Fig. 1.

### Osteotomy of human teeth

Ten teeth were collected as surgical waste during tooth extractions, fixed in formalin, anonymized before they were transported to the laboratory, numbered in chronological order, and osteotomized in the apical part of root. Teeth, which had not been treated within the root region, were used for further investigation. In brief, the tooth was placed flat on the cooled working surface and fixated along the longitudinal axis perpendicular to the working direction of the laser beam. Teeth whose roots could be aligned approximately horizontally to the flat working surface were preferably selected for analysis. Thus, a vertical impingement of the laser beam over an area as large as possible of the laser’s defined application distance was enabled. Several incisions were made per tooth, varying the application time to be able to assess the influence of the application time on potential thermal damage in the same object. This requirement simplified the use of four parallel incisions per tooth with varying application times: two with 4 min (incisions *I*_*1*_ and *I*_*2*_) and two with 8 min (incisions *I*_*3*_ and *I*_*4*_). Technical details of the procedure are summarized in Table 1. In order to create a deep incision, the laser beam was scanned over a tooth surface of 1 mm^2^ using a meander pattern.

**Table 1:** Technical details of the NIRL ablation procedure with 20 Hz repetition rate

Incision No.	Ablation time	Laser shots	Spacing in x and y	Scanning area
*I*_*1*_	4 min	4800	100 μm	2 mm x 0.5 mm
*I*_*2*_	4 min	4800	100 μm	2 mm x 0.5 mm
*I*_*3*_	8 min	9600	100 μm	2 mm x 0.5 mm
*I*_*4*_	8 min	9600	100 μm	2 mm x 0.5 mm

### Thermal imaging camera

Temperature changes during laser ablation of dental roots were recorded with a thermal imaging camera (Seek Thermal Compact, Seek Thermal Inc., Santa Barbara, CA, USA, adjustable focus, field of view: 36°, temperature range: -40°C to 330°C), recording adapted to a smartphone. The camera was used to determine the temperature at the ablation area before and during the laser application. Thereby, a continuous temperature reading with a 9 Hz frame rate was provided. Each ablation scan was separately recorded, and a color-coded temperature scale of the detected range was written into each frame during the recording. After the experiment, the frames corresponding to every two minutes where analyzed for the temperature at at the laser’s point of action to extract representative temperature profiles for all ablations. The local temperature was determined by manually color thresholding of a frame (Fig. 1b,c) using the software Fiji [15], in order to mark the color-code of the ablation area (marked black) and determine its value in the inprinted temperature scale (also marked black).

### Documentation and evaluation of laser ablation

The laser ablation of the root tip was recorded on video using a thermal imaging camera. The appearance of all cuts, i.e., smooth, step-like, irregular, the condition of the cutting bottom, i.e., flat, blasting, and any color changes in the root surface or superficial root layers were visually evaluated throughout the preparation process. After completion of the experiments, each laser-treated tooth was photographed for documentation purposes. Additionally, micro-CT images were taken of all laser cuts, while cavity volumes were determined using optical coherence tomography.

### Micro-computed tomography (μCT)

**Fig. 2:**
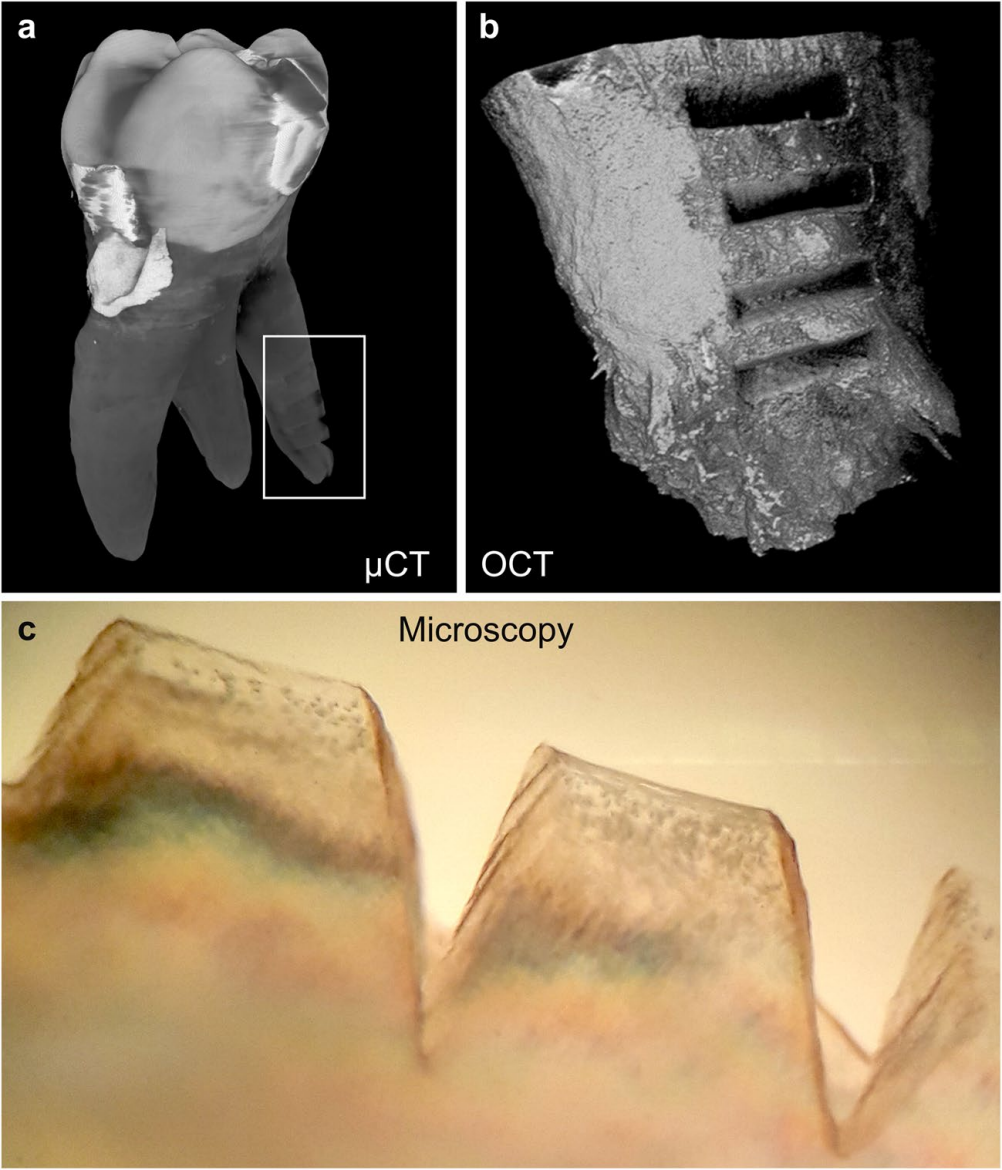
Reconstructed 3D images from the whole tooth imaged with μCT (**a**) and a from the applied laser cuts with OCT (**b**). Microscopy image of the tooth incisions (**c**). The tooth has been halved in the longitudinal direction of the tooth axis and is embedded in Technovit®. The illustration shows the oblique view on the root incisions of the tooth from the cut surface. Parallel grooves are demarcated by dark colored debris on the converging preparation walls

The teeth were further examined using a Skyscan μCT 1272 (Bruker, Kontich, Belgium) operated with a spatial resolution of 7.5 μm. Imaging was performed with a 0.5 mm Al filter, 60 kV acceleration voltage, 166 μA tube current, and 1800 ms exposure time. For analysis, the width, length, and depth of each incision in the root area was imaged and evaluated (Fig. 2a).

### Optical coherence tomography (OCT)

A spectral domain optical coherence tomography system with a central wavelength of 840 nm (OQ Labscope 2.0, Lumedica, Durham, NC, USA) was used for optometric characterization of the ablation surface and volumetry of the cavities (Fig. 2b). The applied OCT imaging volume was 512 × 512 × 512 voxels with each voxel measuring 11.48 × 11.48 × 3.61 μm^3^ in air. The precision of the volumetry was tested by measuring a cavity four times (4.221E+08 μm^3^, standard deviation 1.345E+07 μm^3^). The volume of the ablation sites were manually segmented and quantified with the open-source software ITK-Snap (version 3.8.0) [16].

### Histology

Teeth were cut with a diamond saw (Exakt Advanced Technologies GmbH, Norderstedt, Germany) in the middle of longitudinal axis. One half of the tooth was prepared undecalcified by cutting and grinding the sections to a thickness of approximately 200 μm. Microphotographs of the osteotomy surface were taken of the tooth halves selected for the undecalcified tissue sectioning and embedded in Technovit® to document the three-dimensional structure of the hard tissue removal. The cutting plane allowed the evaluation of the entire depth of incisions caused by the application of NIRL. The transected tooth was processed for histological staining as described elsewhere [14].

### Statistics

Due to a non-parametetric data distribution, the Kruskal-Wallis test was used for all comparisions to determine differences of thermal impact on hard tissue with respect to number of scan applications and ablation depths. A level of significance of 0.05 was chosen.

### Ethics

After informing patients about the study objective, the teeth were voluntarily given to the practitioner for further investigations in all cases. The patients were informed about the study and all of them have given written consent for use of their tissues. These investigations were conducted in accordance with the Hamburgisches Gesundheitsdienstgesetz (Hamburg Health Service Act) and do not require approval by an ethics committee.

## Results

The automated hard tissue ablation was successful in all experiments. No thermal effects, such as smoke formation or discoloration of the edges of the root cuts, were observed during the laser application. The laser was applied for an average of 4 minutes at ablation sites *I*_*1*_ and *I*_*2*_, and for 8 minutes for the sites *I*_*3*_ and *I*_*4*_.

### Heat measurement (infrared camera)

**Fig. 3:**
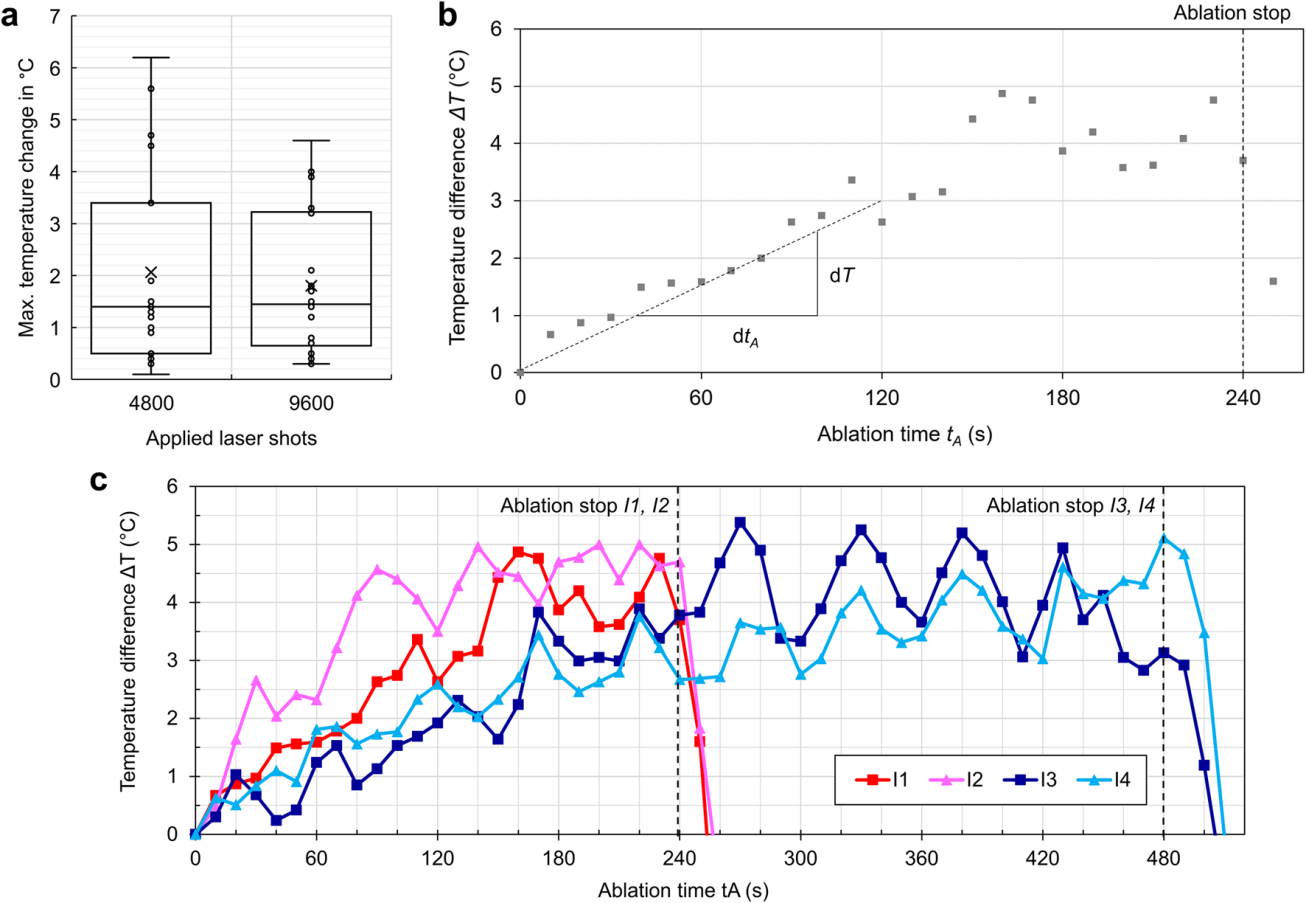
Measurement of NIRL’s thermal effects on dental roots. Maximum temperature change with different numbers of laser shots (**a**). Temperature difference during and after NIRL application on dental roots (**b**). Representative temperature difference in incisions *I*_*1/2*_ terminated after 240 seconds and *I*_*3/4*_ terminated after 480 seconds (**c**)

The heat measurement indicated a rise in temperature during dentin ablation. However, the maximum temperature did not surpass acceptable limits, beyond which permanent biological damage is expected (Fig. 3a,b). The highest temperature difference was measured to be 6.2°C, the mean value was much lower at 1.9°C with a standard deviation of 1.3°C. Although the thermal camera was continuously acquiring images at a 9 Hz frame rate, only a reduced frame set of the ablation was analyzed (each 120 seconds).

To further investigate the temporal dynamics of the temperature profile, the thermal image data of all four incisions (*I*_*1*_ to *I*_*4*_) in one representative tooth was analyzed with a higher resolution of 10 seconds (s. Fig. 3c). After each ablation a very fast temperature drop (~20 seconds) down to the initial temperature was observed.

### Characteristics of cavities

The ablation on the surface and a few millimeters inside the root appeared as a parallel-walled gap. Deeper layers showed cavity walls that deviated at the same angle from the vertical, which was perpendicular to the tooth root surface. These walls converged towards each other, resulting in a narrower ablation area inside the tooth compared to the ablation volume defined close to the root surface. In certain cases, the osteotomy converged to a narrow gap. At the transition from the bottom of the ablation surface to the vertical cutting surfaces, a rounding was consistently observed. However, in some cases, the dentin was removed almost parallel to the bottom of the incision with almost vertical cutting surfaces (Fig. 5b).

### Determination of ablation dimensions with micro-CT

**Fig. 4:**
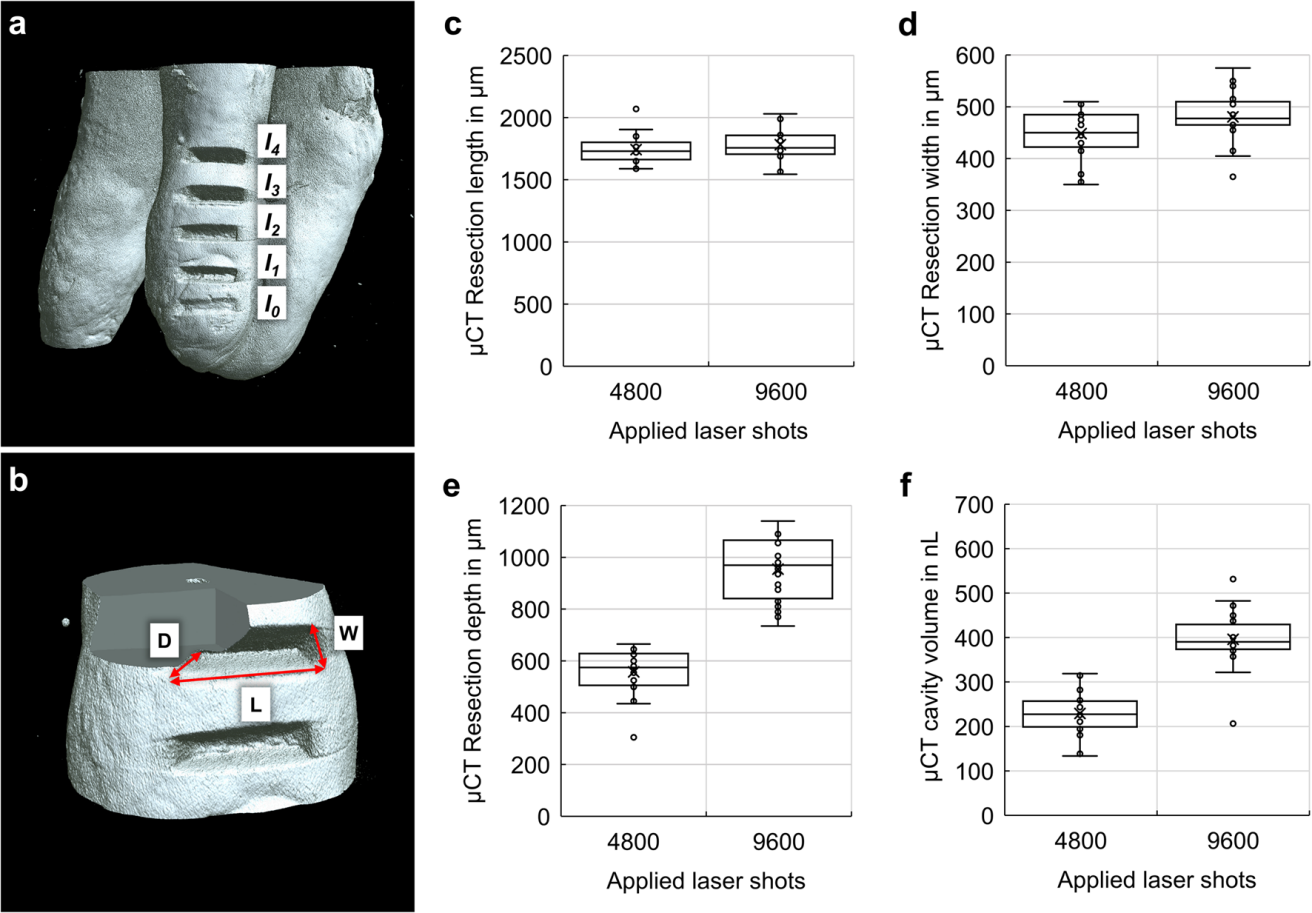
NIRL’s root incisions (**a**) with a test ablation (*I*_*0*_), two times 4800 applied laser shots (*I*_*1*_ and *I*_*2*_) and two times 9600 applied shots (*I*_*3*_ and *I*_*4*_). Measuring the length (L), width (W) and depth (D) of each cavitiy in the μCT images (**b**). The box plots show the determined lengths (**c**), widths (**d**) and depths (**e**) for each of the numbers of shots. Furthermore, the mean cavity volumes were determined (**f**)

The micro-CT software CTAn (Bruker, Kontich, Belgium) was used to measure the length, width, depth and volume of the incision *I*_*1*_ to *I*_*4*_, as shown in Fig. 4a,b. The test ablation site 0 was used to find the right ablation parameters and has therefore been ignored. The following graphs of Fig. 4c-f show the dimensions and volume of all sites over all (*n*=10) measured teeth.

The mean ablation volume of the ablation areas *I*_*1*_ and *I*_*2*_ (4800 laser shots) was 0.229±0.057 mm^3^, resulting in volume of about 48 pL per laser shot. The mean ablation volume of the areas *I*_*3*_ and *I*_*4*_ (9600 laser shots) was 0.396±0.042 mm^3^ with an overall calculated mean volume of 41 pL per shot.

### Calculation of the removed dentin volumes with OCT

**Fig. 5:**
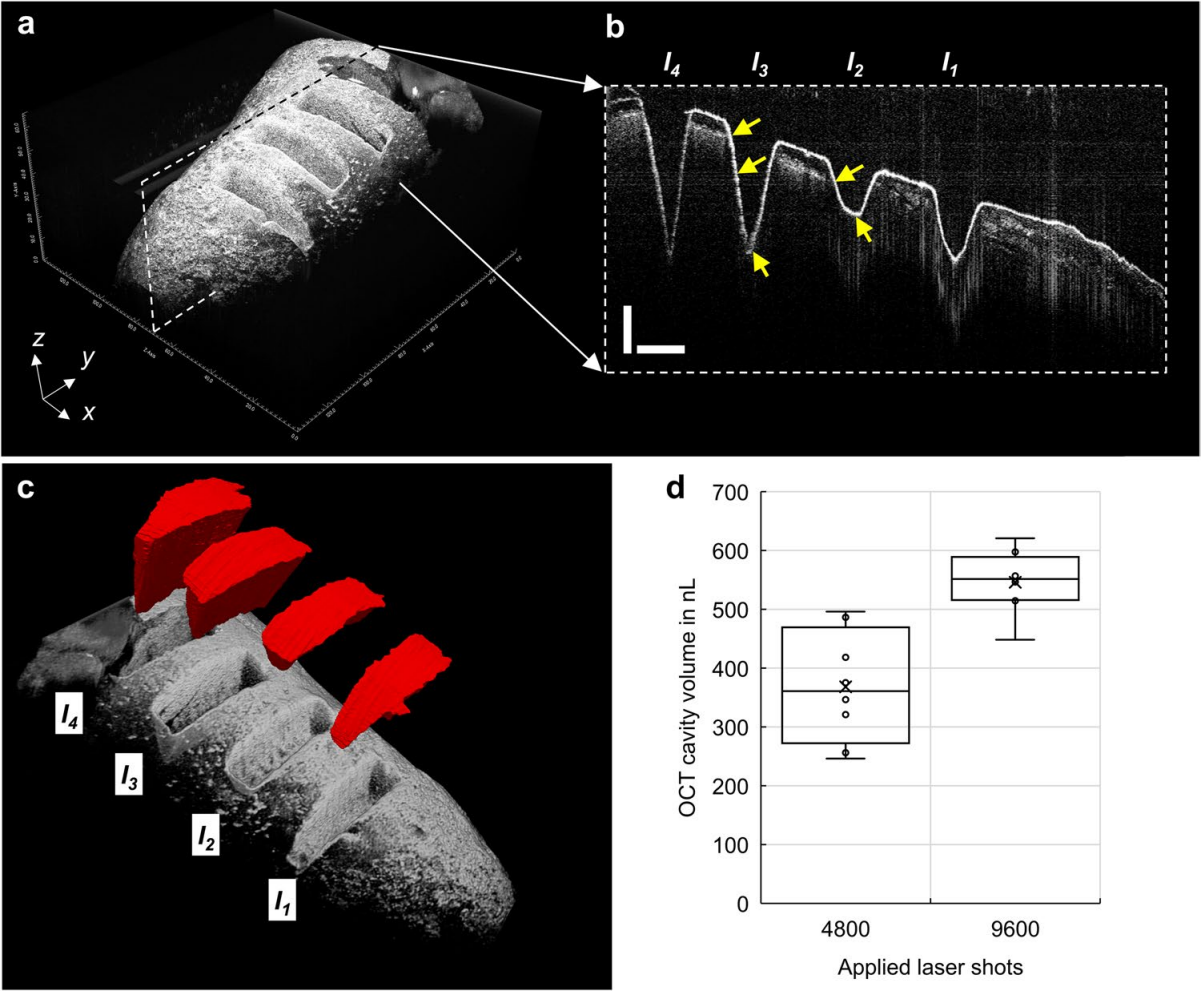
Maximum intensity projection of the four ablation Incisions *I*_*1*_, *I*_*2*_, *I*_*3*_ and *I*_*4*_, image with optical coherence tomography (OCT) (**a**). 3D image data of the tooth root with optical coherence tomography (**a**) and brightness scan (B-scan) of the four ablated cavities (**b**) with a scale bar of 500 μm. The slopes (yellow arrows) are introduced by the shape of the focused laser beam. Ablation volumes for *I*_*1*_, *I*_*2*_, *I*_*3*_ and *I*_*4*_ were manually segmented (red) with the opensource software ITK-Snap [16] (**c**). The box plot shows the quantified volumes for 4800 and 9600 applied laser shots (**d**)

Imaging with OCT was performed on four teeth to complement the μCT measurements and investigate the layered structure of each tooth. OCT revealed the same ablation volumes in the selected teeth like μCT (Fig. 5d).

### Histology

The histological examination confirmed the visual impression of a smooth surface preparation. Thermal effects were not observed either on the surface or in the dentin. The halved specimen already prepared in Technovit® for sawing and grinding was examined using a light microscope. A fine, grooved, dark deposit of debris was apparent (Fig. 1c). These deposits were confirmed in all processed specimens.

## Discussion

This study successfully tested NIRL for precise cutting of tooth roots and found that the experimental setting is effective in preventing significant temperature increase during hard tissue ablation. The results indicate that NIRL is a suitable method for this procedure. The processing residues at the interface of the hard tissue ablation were similar to those after mechanical ablation [17–21]. However, the ablation volume created by NIRL is significantly smaller compared to the voluminous ablation produced by drilling or piezo surgery in this particular indication (Fig. 2c).

Apical osteotomy is an attempt to heal a pulp-dead tooth whose inflammation and necrosis extends beyond the apex causing apical osteolysis. Several mechanical tools are available for a standardized technique which is used to remove the infected root tip to prevent further tooth and bone destruction. The most common tool for hard tissue removal is using a drill. The mechanical ablation causes a shortening of the tooth root. Thus, the treated tooth must adhere to the bone with a reduced contact surface. Mechanical ablations with a drill are an effective and quick way to remove the diseased part of the tooth. However, the ablation properties of the drill are limited in terms of precision due to the diameter of the rotating and ablating working part and shift and deflection of the instrument due to unequal resistance following the mechanical contact with the hard tissue surface. Handling a drill is less gentle on hard tissue than the precise ablation of the root tip by means of a contactless, focused laser beam, in other words, laser ablation offers the option of removing better defined root tip. This characteristic improves the conditions for osseous regeneration by creating a smaller cavity to get re-ossified and a larger root volume remaining in situ with a correspondingly larger contact surface of the root to the bone. The ablation of the root tip with an energy transmission system operated via a piezo element in the ultrasonic range also offers a narrow, defined incision gap which, however, is depending on the device and applicator [22] and is wider than the ablation diameter area of the laser in dentin.

### Ablation precision

The experimental use of the laser for apicoectomy is well documented [13, 23, 24]. The roughness of the ablation surface of lasers in comparison to drill or piezo surgery is evaluated differently in studies depending on the instruments used and the experimental setup, but the assessments concerning the quality of the ablations are similar [14, 22, 25, 26]. All these tools for root tip ablation are considered to have an acceptable quality of the surgically defined root tip surface concerning sealing of the dentin canals, adhesion of sealers, and avoiding chipping or cracking of dentin. This examination describes the wavy relief of the dentin removal at the bottom of the preparation. This pattern in the cavity wall is like the dentin ablation after using a drill or laser [5].

### Thermal alterations

Laser osteotomies do not cause an increase in temperature in the tooth and the surrounding area beyond a level that is physiologically tolerable, provided the osteotomy is sufficiently water-cooled [5, 6]. A rise of 7°C has been considered a limit of the biologically acceptable increase in temperature at the root surface [5, 6]. The use of IR lasers has the technical advantage of emitting energy through very short wavelengths, which destroys molecular compounds at a rate that acts below the time limit allowing thermal reactions [7–9]. However, the potentially athermal transformation of tissues into the gas phase by IR lasers is based on adequate amounts of water [27–30]. Hard tissue requires adequate cooling for osteotomy using laser [27]. Dentin contains less water than bone. Significant increases in temperature during apicoectomy can be the result of imprecise adjustment of the focus as well as overheating of the tissue in a relatively anhydrous environment [14]. The current test results show that NIRL can ablate the tooth root without significant increases in temperature. The comparison with similar studies applying laser for apical root resection of teeth is interesting. A nanosecond pulsed Cr:CdSe laser with a thermal penetration depth was 14.2±0.7 μm at a wavelength of *λ*=2.9 μm showed similar results [5]. In addition, temperature increases with and without cooling were no greater than about 3°C in experimental ablation of enamel using a CO_2_ laser [31]. Indeed, constant addition of fine water mist directly at the ablation side likely will neither substantially decrease the rate and precision of ablation nor cause carbonization and melting in the surrounding dental hard and soft tissue [27].

### Perspectives

Under *in vitro* conditions, the optimal adjustment of the application area and the working beam was achieved by positioning them perpendicularly to each other. The object was also kept constantly cooled during use. In clinical practice, successful transfer of experimental results requires consideration of the different topographical conditions and access paths of the applicator. Indeed, targeted application of the laser beam in apicoectomy can be challenging in the clinical setting, especially in oral surgery for molars. Automated root tip preparation would be an alternative hand-held tools [32, 33]. However, there is currently no routine clinical application of robotic-assisted surgery in apicoectomy [33]. When using a hand-held applicator in clinical routine, it is necessary to develop a flexible, angulable handpiece that allows for free application of the laser beam in the oral cavity. Current developments of a flexible applicator have to consider clinical requirements [34]. However, using IR lasers (NIRL or PIRL) with a wavelength of 2940 nm and integrated into a handheld still leaves some technical issues to solve. In order to allow safe application in the oral cavity, the applicator has to be very small, ideally equipped with a beam scanning mechanism and a depth control. Initial results have shown that multimode fiber can provide lossless energy transfer of IR laser to difficult-to-reach objects [35]. Furthermore, an integrated control of the temperature in the application area is required in view of the high energy that impinges on biological units at certain points.

At present, the long application time during experimental use of the laser is still unsuitable for clinical use. Therefore, it is essential to increase the repetition rate of the NIRL while asuring a spatial distance between each laser shot, e.g. by randomized sampling during a line scan, for clinical applications. These technical improvements would significantly advance this laser application in oral surgery (and endodontics) towards clinical applicability. In addition, these further developments bring the treatment of larger hard tissues within the reach of clinical applications, for example osteotomy of bones.

## Conclusion

Lasers with a very high wavelength such as the NIRL allow a nearly athermic dissociation at the subcellular level in soft tissues. When used in teeth, thermal effects occur. With the NIRL application in these tissues, the comparatively lower ablation rate is probably a consequence of the energy applicable to this setting and can therefore be technically modified. The low water content of the object was not a factor limiting the application, because the temperatures under the laser osteotomy remained within the physiologically tolerable range. Faster scanning with higher power NIRLs and a mobile applicator adapted to the oral conditions are prerequisites for clinical use. However, the precision of the incisions in dentin is excellent. Expected technical optimizations of NIRL offer an alternative to current tools for apical root resection.
